# The Efficacy and Safety of a Herbal Toothpaste in Reducing Gingivitis: A Double-Blind, Randomized, Placebo-Controlled, Parallel Allocation Clinical Trial

**DOI:** 10.1155/2019/3764936

**Published:** 2019-02-03

**Authors:** Jinfeng He, Yalan Deng, Fangzhi Zhu, Ting Zhong, Nanyu Luo, Lei Lei, Li Cheng, Tao Hu

**Affiliations:** State Key Laboratory of Oral Diseases & National Clinical Research Center for Oral Diseases & Department of Preventive Dentistry, West China Hospital of Stomatology, Sichuan University, Chengdu, China

## Abstract

**Aim:**

To examine the efficacy and safety of the toothpaste containing Rhizoma Chuanxiong and Rhizoma Imperatae extracts in reducing gingivitis.

**Method:**

A double-blind clinical trial was conducted, in which 120 volunteers were randomly assigned to the test group (*N* = 60) or the control group (*N* = 60). Tetramethylpyrazine, senkyunolide A, ferulic acid, and ligustilide are the main effective components of Rhizoma Chuanxiong and Rhizoma Imperatae contains the main components of cylindrin, carotene, 5-hydroxytryptamine, potassium, and calcium. The control group used placebo toothpaste containing neither Rhizoma Chuanxiong extract nor Rhizoma Imperatae extract. Plaque, gingivitis, and bleeding were assessed at the baseline, prior to the supragingival scaling, and at 4, 8, and 12 weeks.

**Results:**

During the trial, both test and control groups showed a decreasing trend compared to the baseline. At the end of 12 weeks, with respect to Gingival Index (GI), Bleeding Index (BI), and Bleeding on Probing percentage (BOP%) scores, there were significant differences between test and control groups (GI,* P*<0.001, BI,* P*<0.001, and BOP%,* P*<0.001, resp.). After 4 weeks of usage, there were no statistically significant differences in all of GI, BI, and BOP% scores between the two groups. However, the decrease became statistically significant at next two intervals (GI,* P*<0.001, BI,* P*<0.001, and BOP%,* P*<0.001, resp.) in the efficiency of GI, BI, and BOP% which was 8.04%, 11.02%, and 37.16%, respectively. There were no treatment-related adverse events reported.

**Conclusion:**

The toothpaste containing Rhizoma Chuanxiong and Rhizoma Imperatae extracts was well tolerated and significantly reduced gingivitis and bleeding after usage for 12 weeks. There was better improvement at molars, and the more serious the baseline status was, the better the efficacy was.

## 1. Introduction

Chronic gingivitis is one of the most common oral diseases with high prevalence around the world [1]. A survey of the prevalence and severity of gingivitis in American adults shows that the prevalence of gingivitis among adults ranged from 56% to 94% [2]. Even though the factor causing gingival lesions to be converted into periodontitis has not been well understood, current theory holds that the gingival lesion is the precursor of periodontitis [3]. Recent studies have also found that gingivitis is associated with a number of systemic diseases [4]. Therefore, the prevention and elimination of gum inflammation are essential for maintaining oral health and overall health [5].

Dental plaque is the major etiological and initiating factor for the development of gingivitis [6]. Therefore, the ideal plaque control is the basis for the prevention and control of gingivitis. It is considered that individual continuous removal of dental plaque is the most effective means of preventing and controlling gingivitis, in which brushing teeth and other mechanical methods to remove plaque are generally recognized as effective strategies [7]. However, due to the limitation of mechanical methods, the addition of some safe and effective drugs to prevent gingivitis in toothpaste is also considered to be a good supplementary to the control of mechanical plaque [8–10]. Studies have shown that certain chemicals, such as chlorhexidine or triclosan, are added to the toothpaste to directly inhibit the formation of plaque [11, 12], nevertheless with the side effects of antimicrobial resistance, teeth coloring, taste changes, and so on [11, 13]. Recently, Chinese herbal medicinal ingredients have become the focus of research because of their natural, relatively low toxicity and cultural background [14–16].

**Figure 1:**
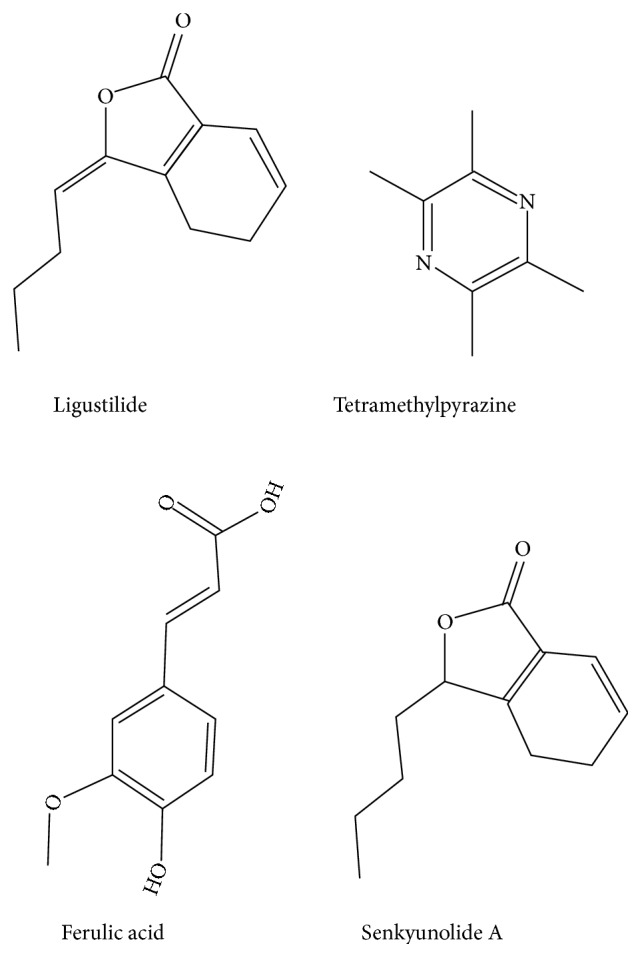
Chemical structures of main active monomers in Rhizoma Chuanxiong [19].

Rhizoma Chuanxiong is the dried rhizome of* Ligusticum chuanxiong* Hort, known as a famous medicinal herb from Sichuan, and its main components include volatile oil, acid composition, and nitrogenous compounds [17]. In the past 50 years, pharmaceutical researchers have carried out a large number of studies on the pharmacodynamic basis of Rhizoma Chuanxiong. About 174 compounds have been isolated and identified from this herb [18]. The main effective components of Rhizoma Chuanxiong include tetramethylpyrazine, senkyunolide A, ferulic acid, and ligustilide [19]. Chemical structures of monomers are reported in Figure 1 [19]. Traditional Chinese medicine holds that Rhizoma Chuanxiong has the effect of invigorating blood and expelling wind-damp and relieving pain [20, 21]. Rhizoma Chuanxiong has been commonly used as traditional medicine for the treatment of various kinds of diseases including cerebrovascular diseases, migraine, maxillary sinusitis, pharyngitis, arthritis, and nephritis [22, 23]. However, the applications regarding Rhizoma Chuanxiong in the field of oral medicine have been poorly understood, especially in the prevention and treatment of periodontal diseases.

Rhizoma Imperatae is the rhizome of* Imperata cylindrica *(L.) Beauv. var.* major *(Nees) C.E.Hubb., which is a common perennial grass [24, 25]. As a traditional Chinese herb, Imperata Rhizoma has been reported to have the functions of clearing heat and cooling blood, as well as hemostatic effect [26]. The main components of Rhizoma Imperatae include cylindrin, carotene, 5-hydroxytryptamine, potassium, and calcium. Modern pharmacological research shows that Rhizoma Imperatae mainly has functions of hemostatic, diuresis antihypertensive, bacteriostasis, anti-inflammatory, analgesic, and anti-tumor, as well as reducing hydroxyl radical, antioxidant, and enhancing immunity [26, 27]. At present, there is a potential use of its hemostatic principle, and the Rhizoma Imperatae extract has been added to oral care products, which shows excellent effect on reducing gingival bleeding [28].

According to Chinese Pharmacopoeia (2015 edition), the rhizomas of* Ligusticum chuanxiong* Hort and* Imperata cylindrica *(L.) Beauv. var.* major *(Nees) C.E.Hubb. are the main medicinal parts and have been commonly used in traditional medicines and even added to the diet. Rhizoma Chuanxiong has been recorded on the herbal list declared by Ministry of Health of China, in which 101 herbs can be used for medicinal drugs and health products. In addition, the material from the toothpaste producer shows that the effective components of rhizomes of Chuanxiong and Imperatae are higher than those of stems and leaves, and the medicinal value is also greater. Therefore, the rhizomes were chosen to make the extract.

The compatibility of Rhizoma Chuanxiong and Rhizoma Imperatae is also used in traditional medicine. It has been reported that this compatibility can promote blood circulation, protect the vasculature, and inhibit bacteria, so as to achieve the effect of hemostasis, anti-inflammation, pain relief, and so on [29]. Taken together, the compatibility of Rhizoma Chuanxiong and Rhizoma Imperatae indicates they could be potentially used in the prevention and treatment of gingival bleeding, gingivitis, and periodontitis. The purpose of this study was to investigate the clinical efficacy and safety of Chinese herbal toothpaste containing the extracts of Rhizoma Chuanxiong and Rhizoma Imperatae in supragingival plaque formation and gingivitis progress when compared to placebo toothpaste over a period of 3 months.

## 2. Materials and Methods

### 2.1. Study Design and Population

A 12-week randomized, double-blind, placebo-controlled clinical trial was conducted at West China Hospital of Stomatology, Sichuan University, Chengdu, China. The study protocol was approved by the institutional ethical board at West China College of Stomatology, Sichuan University (WCHSIRB-D-2017-078), and was in good accordance with the World Medical Association Declaration of Helsinki on ethical aspects and related regulations for clinical studies in China. This trial was registered as a clinical study (registration number: ChiCTR1800015742).

All participants in the study were volunteers. All voluntary participants were informed of the outline, purpose, and duration of the study and signed an informed consent form before enrolment. 120 participants were enrolled in this clinical research based on the inclusion and exclusion criteria.

#### 2.1.1. Inclusion Criteria

To be included in the trial, volunteers must be

(i) aged 18 to 70 years, male and female,

(ii) of good general health, having daily tooth-brushing habit,

(iii) possessing >20 natural permanent teeth that are uncrowned and at least 5 natural teeth in each quadrant,

(iv) diagnosed with gingivitis, GI ≥ 1 in 60% of the sites according to the Loe-Silness GI, having a whole-mouth mean PI ≥ 1.0 according to the modified Quigley and Hein index,

(v) signing the written consent before the initiation of the study and completing this clinical trial as required.

#### 2.1.2. Exclusion Criteria

(i) Advanced periodontal disease, pulpitis, or open caries or soft tissue lesions

(ii) Allergy to the study product components

(iii) Usage of antibiotics, anticoagulant drugs, anti-inflammatory medication, or other drugs that may affect the results of the trial within the preceding 1 month

(iv) Wearing orthodontic bands or partial or removable dentures

(v) Pregnancy or breast-feeding

(vi) Receiving oral prophylaxis in the past 2 weeks

(vii) Participation in other similar tests in the past 3 months

#### 2.1.3. Treatment Method

The toothpaste used in the both study groups was produced by Sichuan Green Herb Technology Development Co., Ltd. (Century City South Road, Hi-Tech Zone, Chengdu, Sichuan, China). The experimental toothpaste contained Rhizoma Chuanxiong extract, Rhizoma Imperatae extract, sorbitol, hydrated silica, water, glycerin, polyethylene glycol 400, sodium lauryl sulfate, carrageenan, xanthan gum, hydroxyethyl cellulose, saccharin, sodium benzoate, pigment CI42053, and edible saccharin. The extracts were extracted from the rhizomes by decocting method. 40-60 pieces of Rhizoma Chuanxiong and 35-70 pieces of Rhizoma Imperatae were mixed and crushed; after being heating-extracted by distilled water, the mixture was extracted by vacuum filtration. The extract was concentrated, vacuum-frozen, or spray-dried, and the traditional Chinese medicine composition with hemostatic, anti-inflammatory, and antibacterial effects was obtained. The best formulation was obtained by screening different formulations, and the results were verified by pharmacodynamic tests, so as to select the prescription.

Through random codes produced by SAS software, eligible subjects were block-randomized into the test group or the control group; allocation ratio was 1:1. Each group comprised 60 people. The baseline data were recorded and each participant was given a thorough scale using ultrasonic instruments to remove supragingival plaque. Then every patient was provided with the assigned toothpaste and the same adult soft-bristled toothbrush. The placebo toothpaste contained the same ingredients as the experimental toothpaste except for Rhizoma Chuanxiong extract and Rhizoma Imperatae extract. Toothpaste was in identical tubes; the labels were not revealed until the end of the study. Toothpaste was dispensed by an investigator not involved with clinical examinations. The participants were also not aware of the type of the toothpaste allocated to them. Every four weeks, the subjects were provided the new toothpaste and toothbrush according to number, and the used toothpaste would be collected. All participants who refrained from all other oral hygiene procedures during the study period were given professional brushing instructions and instructed to brush their teeth twice daily for 2-3 minutes.

At baseline, 4 weeks, 8 weeks, and 12 weeks, patients came to the clinical research center and were examined. All oral hygiene practices, such as brushing, flossing, and mouth-rinsing, were prohibited for 12 hours before examinations. Eating, drinking, and smoking were also prohibited for 4 hours before examinations. For clinical examinations, participants were instructed to refrain from brushing for about 12 hours before the clinic visits.

The examination was performed with CPI probe in the same clinical room. At baseline and the subsequent visits, three indicators were used to assess clinical efficacy. The Bleeding Index (BI) [30, 31] and the Gingival Index (GI) [32] were performed to assess the inflammatory state of the gums by an investigator. Then the Turesky modification of the Quigley-Hein Plaque Index (PI) [33, 34] was performed under the assistance of plaque indexes (GERMIPHENE, USA) to evaluate dental plaque by other investigators.

Safety observation indexes include (1) vital signs, such as blood pressure, respiration, body temperature, and heart rate, (2) adverse events and/or reactions that may occur, focusing on the presence of allergies and irritation (lips, buccal, tongue, and other soft tissue conditions), nausea, and so forth, and (3) suitability indicators of taste and tolerance.

### 2.2. Statistical Analysis

The therapeutic effect was evaluated by per-protocol set (PPS), and safety data set (SS) was used for safety evaluation. The management of the data was performed using the SAS 9.2 statistical program.

## 3. Results

**Figure 2:**
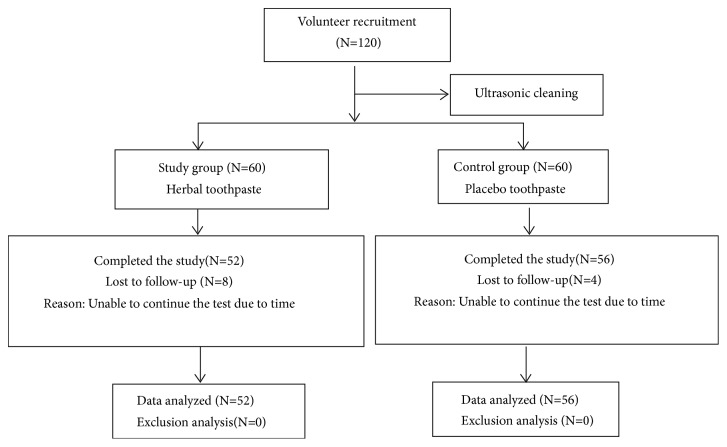
Flow of participants through each stage of the trial.

According to the formula n1=n2 = 2[(Z_*α*/2_+Z_*β*_)S/*δ*]^2^ [35], considering the loss rate at 20%, sampling error, our manpower, and material resources, also referring to the standards of health industry of China and other toothpaste trails [36, 37], 120 participants eventually entered clinical trial and were equally allocated to the experimental group and the control group. A total of 108 cases entered the final statistical analysis, including 52 patients in the test group and 56 patients in the control group. The subjects failed to complete the study for some reason, which has nothing to do with the dentifrices. The study flow diagram is shown in Figure 2.

There were no statistically significant differences neither in age nor in gender between the two groups. There were also no statistically significant differences on PI, GI, BI, or BOP% between the two groups (Table 1), which demonstrates that the group assignment was appropriate.

During the trial, both test and control groups showed a decreasing trend compared to the baseline. At the end of 12 weeks, with respect to GI, BI, and BOP% scores, there was a significant difference between test and control groups (GI,* P<*0.001, BI,* P*<0.001, and BOP%,* P*<0.001, resp.). However, the differences between two groups were not statistically significant with respect to all indexes at the 4-week and 8-week time intervals and the PI score between test and control groups at any time interval (Table 2).

The reduction percentage (mean ± Std.) for all groups and all parameters are given in Table 3. The difference between two groups was statistically significant with respect to GI, BI, and BOP% by the 8-12-, 4-12-week time intervals, and the reduction percentage of the test group was greater than that of the control group. However, there was no significant difference between test and control groups with respect to PI scores.

It was reported that there were significant variations in plaque accumulation within dentition, and tooth position within the alveolar bony strongly correlated with gingival recession [38, 39]. The efficiency differences among tooth types were evaluated when the trial was finished (Table 4). All three indexes were normally distributed (*P*>0.05) and their variances were also homogeneous (*P*>0.1). The results of ANOVA showed that there were significant differences between molar and premolar or anterior teeth with respect to GI and BI scores.

**Figure 3:**
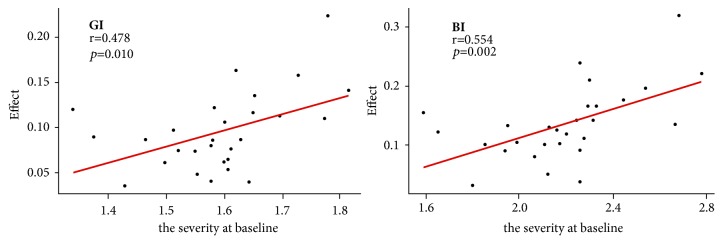
The correlation between effect of toothpaste and the severity at baseline. Efficiency = (control-test)/control*∗*100%.

Furthermore, it was noticed that the severity at baseline throughout dentition varied at baseline examination. As a result, the correlation between the effect of toothpaste and the degree of gingivitis at baseline was examined, and significant positive correlation was found with respect to GI (r=0.478,* P*=0.010) and BI (r=0.554,* P*=0.002) scores, revealing that the more serious the baseline status, the better the effect of toothpaste (Figure 3).

As strict principles were undertaken, 42 cases of adverse events were reported, which included 25 cases in the control group and 17 cases in the test group. However, the researchers determined that their relationship with the use of toothpaste was “probably irrelevant” and not correlated with toothpaste adverse reactions. There was no statistically significant difference in the compliance (%) between the two groups of subjects (Table 5).

No bad/terrible-taste or hard-to-accept was reported in both groups, and the difference of the constituent ratio between two groups was not statistically significant with respect to taste and tolerance degrees (*P*=0.648,* P*=0.829).

## 4. Discussion

In the current study, the toothpaste containing the herbal ingredients Rhizoma Chuanxiong and Rhizoma Imperatae was tested for its efficacy and safety during 12 weeks of twice daily use in improving gingival and oral hygiene.

After 4 weeks of use, there were no statistically significant differences in the reduction of all parameters between the two groups (*P*>0.1). However, the reductions became statistically significant in the next two intervals (GI,* P*<0.001, BI,* P*<0.001, and BOP%,* P*<0.001, resp.). The efficiency of GI, BI, and BOP% was 8.04%, 11.02%, and 37.16%, respectively. The results demonstrated that the herbal toothpaste was effective in inhibiting gingivitis and oral health maintenance when compared to the negative control toothpaste. Moreover, a better efficacy was also found in the molars than the premolars and the anterior teeth. The effect has strong correlation with the severity at baseline with respect to GI (r=0.478,* P*=0.010) and BI (r=0.554,* P*=0.002) scores; the more serious the baseline status is, the better the effect is. It is indicated that more serious gingivitis may achieve a better improvement when treated with herbal toothpaste.

As an authentic herbal medicine of Sichuan, Rhizoma Chuanxiong was first recorded in the Divine Husbandman's Classic of the Materia Medica (*Shen Nong Ben Cao Jing*) more than 400 years ago. In traditional Chinese medicine beliefs, Rhizoma Chuanxiong has the effect of invigorating blood and expelling wind-damp as well as relieving pain. During the past years, valuable information has been obtained on its pharmacology. Rhizoma Chuanxiong mainly contains organic acid, essential oils, and nitrogenous compounds. Tetramethylpyrazine, senkyunolide A, ferulic acid, and ligustilide are the main effective components [19]. Their main pharmacological effects include removal of oxygen free radical, antibiosis and anti-inflammatory, which was reported to increase of immune function, anti-platelet and can promot blood circulation, etc [40–46]. For anti-inflammatory activity, the modern pharmacological research discovered that Z-ligustilide and senkyunolide I exerted a potential anti-inflammatory effect on microglia through inhibition of NF-kappa B pathway [41, 47, 48]. Another study showed that the Rhizoma Chuanxiong essential oil fraction, senkyunolide H and senkyunolide O, inhibited significantly the production of nitric oxide and proinflammatory mediators such as IL-1*β*, IL-6, and TNF-*α* and also reduced the expression levels of cyclooxygenase-2 (COX-2) as well as inducible nitric oxide synthase (iNOS) [45, 49, 50]. For antioxidant activity, Z-ligustilide was reported to show a comprehensive antioxidant effect on the spontaneous oxidation of linoleic acid, mitochondrial oxidation, homogenate spontaneous oxidation, and oxidation induced by H_2_O_2_ [51]. Besides, tetramethylpyrazine was reported to significantly remove free radicals, effectively alleviate the oxidative stress, and decrease the reactive oxygen species formation induced by gentamicin [52, 53]. In addition, it was reported that senkyunolide H and senkyunolide I possibly attenuated oxidative damage by activating the HO-1 pathway and enhanced the cell resistance to oxidative damage related to hydrogen peroxide [54]. The blood circulation improvement effect of Rhizoma Chuanxiong was reported to be related with tetramethylpyrazine because it ameliorated platelet activation, aggregation, and adhesion. This procedure induced sustained infiltration and activation of various inflammatory cells, including lymphocytes and eosinophils [46].

Rhizoma Imperatae is also a common Chinese herbal medicine, of which the main components include cylindrin, carotene, 5-hydroxytryptamine, potassium, and calcium. Modern pharmacological research shows that Rhizoma Imperatae mainly has functions of hemostatic, diuresis, antihypertensive, bacteriostasis, anti-inflammatory, analgesic, and antitumor, reducing hydroxyl radical, antioxidant, and enhancing immunity [26, 27]. The extract of dry* Imperata cylindrica* (L.) was reported to show antimicrobial activity on* Escherichia coli*,* Bacillus subtilis*,* Pseudomonas aeruginosa*, and* Staphylococcus aureus* [55]. The methanol extracts from the leaves of* Imperata cylindrica* (L.) were reported to show the anticancer activity in human oral cancer cell line [56]. Isoeugenin revealed the anti-inflammatory effects on LPS-activated RAW264.7 macrophages by inhibiting nitric oxide (NO) formation [57]. The polysaccharides from Rhizoma Imperatae showed high antioxidant activity including hydroxyl radical scavenging activity and 2,2-diphenyl-beta-picrylhydrazyl radical scavenging activity [58]. At present, there is a potential use of its hemostatic principle, and the Rhizoma Imperatae extract has been added to oral care products, which showed excellent effect on reducing gingival bleeding [28].

Existing theories show that Rhizoma Chuanxiong companied with Rhizoma Imperatae has great application potential and value in the treatment of gingivitis. The efficacy of the variety of Chinese herbal toothpaste has been studied previously. However, there are few standardized clinical studies on Rhizoma Chuanxiong or Rhizoma Imperatae regarding the extract-containing toothpaste at present. The results from the current clinical study could not be directly compared with similar studies. And this is the first study reported in the medical literature which explores the effects of toothpaste containing Rhizoma Chuanxiong and Rhizoma Imperatae extracts on dental plaque and gingivitis. Effects of herbal toothpaste on reducing plaque formation and gingival inflammation were investigated in clinical studies and results were equivocal, which demonstrated significant plaque reductions ranging from 7.17% to 61.2% and gingivitis reductions ranging from 5.20% to 70.6% [59–61].

When the study ended, both types of toothpaste were effective in decreasing plaque and gingivitis parameters compared with the baseline. And the experimental group showed a decreased trend, while the control group went up at the end of 12 weeks. The reduction in indexes in the control group may be attributed to ultrasonic cleaning and repeated oral education, which probably improve oral health a lot. However, this influence was temporary, and the herbal toothpaste can help maintain this improvement. Moreover, the current study showed a better improvement in the molars. And the more serious the baseline status is, the better the effect is. It could be speculated that the molar is not easy to be cleaned resulting in worse health condition, and the herbal toothpaste has a broad application prospect in the treatment of gingivitis, especially severe inflammation. The reduction of dental plaque between test and control groups has no significant difference, while the bleeding improved a lot in test group. These phenomena may be attributed to the anti-inflammatory blood circulation, which improves the functions of Rhizoma Chuanxiong and Rhizoma Imperatae; even no obvious antibacterial effect was observed or reported. All of these may provide the theoretical basis of promoting blood circulation and removing stasis in traditional Chinese medicine. In addition, the observed effect in present study may be attributed to participants' awareness of enrolling in oral hygiene study—Hawthorne effect, no matter what toothpaste they receive. Therefore, extending observation time to a longer period will provide a more powerful comparison.

As the placebo-controlled setting can minimize the subjective expectation effect and bias of subjects and researchers and directly measure the difference in efficacy and safety between the test drug and placebo, it can give the appropriate conclusion of the test drug with a smaller sample. For the positive control, there is little difference between the efficacy of the test drug and that of the positive control drug, so a larger sample is needed to achieve the same efficacy in order to detect the difference between the two drugs. It would be better to set both placebo control and positive control, but, considering our study purpose, the test conditions, manpower, and material resources, only placebo control was chosen, which was the limitation of our study. We would add “positive control” group in our similar clinical trials in the future.

## 5. Conclusion

Within the limits of this clinical study, regular use of the herbal toothpaste containing Rhizoma Chuanxiong and Rhizoma Imperatae extracts could effectively and safely reduce gingivitis of the study subjects. There is a better improvement at molars, and the more serious the baseline status is, the better the effect is. Further long-term studies are needed to confirm its benefits.

## Figures and Tables

**Table 1:** Demographic data and examination status at the baseline.

Index		Control	Test	P value

Gender	Male	20 (35.7%)	20 (38.5%)	0.768 ^a^
n (%)	Female	36 (64.3%)	32 (61.5%)	

Age ($\overset{-}{X}$±SD)		40.91±13.16	36.94±13.43	0.174 ^b^

PI ($\overset{-}{X}$±SD)		2.66±0.54	2.69±0.47	0.797 ^c^

GI ($\overset{-}{X}$±SD)		1.58±0.15	1.61±0.17	0.371 ^c^

BI ($\overset{-}{X}$±SD)		2.15±0.35	2.24±0.43	0.252 ^c^

BOP (%) ^d^($\overset{-}{X}$±SD)		58.58±14.46	61.32±16.98	0.369 ^c^

^a^Chi-squared test.

^b^Wilcoxon rank-sum test.

^c^Independent samples *t* test.

^d^BOP% = bleeding sites on probing/total sites × 100%.

**Table 2:** PI, GI, BI, and BOP% at follow-up.

Index	Time point	Group	Mean	P25	P50	P75	Std.	Mean efficiency ^c^	*P* value

Pi	4 weeks	Control	2.37	1.92	2.35	2.75	0.62	1.27%	0.411 ^a^
		Test	2.40	1.97	2.45	2.70	0.48		
	8 weeks	Control	2.27	1.91	2.35	2.71	0.60	4.85%	0.170 ^a^
		Test	2.38	1.93	2.34	2.83	0.52		
	12 weeks	Control	2.29	1.92	2.21	2.63	0.57	3.49%	0.232 ^a^
		Test	2.37	2.04	2.42	2.73	0.48		

GI	4 weeks	Control	1.12	1.03	1.10	1.16	0.12	3.57%	0.190 ^b^
		Test	1.16	1.04	1.12	1.24	0.15		
	8 weeks	Control	1.07	1.00	1.05	1.10	0.09	12.15%	0.487 ^b^
		Test	1.06	1.00	1.06	1.11	0.16		
	12 weeks	Control	1.12	1.04	1.10	1.19	0.10	-8.03%	<0.001 ^*∗*b^
		Test	1.03	0.96	1.00	1.08	0.10		

BI	4 weeks	Control	1.20	1.05	1.15	1.27	0.19	4.17%	0.309 ^b^
		Test	1.25	1.07	1.18	1.36	0.25		
	8 weeks	Control	1.10	1.01	1.08	1.15	0.13	-0.91%	0.414 ^b^
		Test	1.09	1.00	1.08	1.15	0.11		
	12 weeks	Control	1.18	1.06	1.15	1.28	0.15	-11.02%	<0.001 ^*∗*b^
		Test	1.05	0.97	1.01	1.12	0.13		

BOP%	4 weeks	Control	14.36	5.80	11.65	20.23	10.92	22.14%	0.199 ^b^
		Test	17.54	7.44	12.66	24.01	14.46		
	8 weeks	Control	9.55	4.32	7.14	12.79	8.03	-2.09%	0.409 ^b^
		Test	9.35	3.72	8.33	12.50	6.71		
	12 weeks	Control	15.61	8.48	14.88	21.40	8.76	-37.16%	<0.001 ^*∗*b^
		Test	9.81	4.43	7.71	12.50	7.79		

*∗* indicates statistical significance.

^a^Independent samples *t* test.

^b^Wilcoxon rank-sum test.

^c^Mean efficiency = (test - control)/control *∗* 100%.

**Table 3:** Percentage reductions in clinical indices at every follow-up.

Index	Time point	Group	Mean	P25	P50	P75	Std.	Mean difference ^c^	*P* value

PI	8 weeks -4 weeks	Control	-3.88	-11.27	-7.00	4.69	13.47	3.24	0.105 ^a^
		Test	-0.67	-8.41	-3.96	6.64	12.90		
	12 weeks -8 weeks	Control	2.44	-7.04	2.97	12.18	13.64	-1.53	0.286 ^a^
		Test	0.91	-9.40	-2.08	11.96	14.33		
	12 weeks -4 weeks	Control	-2.22	-10.90	-4.25	7.74	14.96	1.28	0.307 ^a^
		Test	-0.94	-6.97	-1.73	4.56	11.18		

GI	8 weeks -4 weeks	Control	-4.41	-8.06	-4.14	1.61	7.30	-3.37	0.011 ^a^
		Test	-7.78	-14.22	-6.91	-1.27	7.68		
	12 weeks -8 weeks	Control	5.26	1.73	3.91	8.16	5.73	-8.55	<0.001 ^*∗*b^
		Test	-3.28	-7.87	-3.99	0.60	6.03		
	12 weeks -4 weeks	Control	0.42	-4.62	0.28	3.91	7.16	-11.46	<0.001 ^*∗*b^
		Test	-11.04	-15.46	-10.95	-5.83	6.70		

BI	8 weeks -4 weeks	Control	-6.44	-11.85	-4.93	-1.54	9.30	-4.04	0.019 ^a^
		Test	-10.50	-20.87	-9.65	-1.60	10.57		
	12 weeks -8 weeks	Control	7.44	2.23	5.47	11.71	8.34	-11.49	<0.001 ^*∗*b^
		Test	-4.05	-8.42	-5.17	0.00	6.67		
	12 weeks -4 weeks	Control	0.18	-6.63	0.84	6.88	9.63	-14.57	<0.001 ^*∗*b^
		Test	-14.39	-22.27	-13.36	-7.11	9.52		

BOP%	8 weeks 4 weeks	Control	-20.62	-65.68	-33.33	5.69	60.43	-7.78	0.257 ^b^
		Test	-28.40	-59.02	-43.65	-8.57	53.41		
	12 weeks -8 weeks	Control	158.66	21.37	68.11	167.80	231.47	-118.81	<0.001 ^*∗*b^
		Test	39.85	-31.26	8.11	57.50	124.37		
	12 weeks -4 weeks	Control	48.33	-18.97	15.14	67.17	96.18	-73.14	<0.001 ^*∗*b^
		Test	-24.81	-62.15	-40.27	-1.56	59.45		

*∗* indicates statistical significance at p<0.05.

^a^Independent samples *t* test.

^b^Wilcoxon rank-sum test.

^c^Mean difference = test – control.

**Table 4:** Efficiency differences between tooth types at 12 weeks ^a^.

Index	Tooth types	Mean efficiency ^b^	Efficiency difference ^c^	*P* value

GI	Premolar	8.7%	-5.12%	0.013 ^*∗*^
	Molar	13.8%		
	Anterior	7.1%	-6.73%	<0.001 ^*∗*^
	Molar	13.8%		
	Anterior	7.1%	-1.61%	0.546
	Premolar	8.7%		

BI	Premolar	12.6%	-7.83%	0.003 ^*∗*^
	Molar	20.5%		
	Anterior	9.6%	-10.88%	<0.001 ^*∗*^
	Molar	20.5%		
	Anterior	9.6%	-3.04%	0.291
	Premolar	12.6%		

*∗* indicates statistical significance at p<0.05.

^a^ANOVA.

^b^Mean efficiency = (test - control)/control *∗* 100%.

^c^Efficiency difference means the difference between two groups.

**Table 5:** Comparison of adverse events and compliance (SS) ^a^.

Index		Test	Control	*P* value

Adverse event	No	42 (71.2%)	33 (56.9%)	0.107
	Yes	17 (28.8%)	25 (43.1%)	
<80%		1 (1.9%)	3 (5.4%)	0.664
80%~120		51 (98.1%)	53 (94.6%)	

^a^Chi-squared test.

## Data Availability

The data used to support the findings of this study are included within the article.

## Supplementary Materials

Supplementary Materials In the Supplementary Materials, we present the adverse events report which could not be included in the manuscript because of space limitations. The adverse events report showed the details of every adverse event reported including the description, duration, and any treatment undertaken. The relationship between the adverse events and the use of toothpaste was confirmed to be “probably irrelevant” and not correlated with toothpaste adverse reactions.
