# Supplementary material for: The Efficacy and Safety of a Herbal Toothpaste in Reducing Gingivitis: A Double-Blind, Randomized, Placebo-Controlled, Parallel Allocation Clinical Trial
Source: Evid Based Complement Alternat Med. 2019 Feb 3;2019:3764936. doi: 10.1155/2019/3764936 (PMC6378009; doi:10.1155/2019/3764936)
Supplement: Supplementary Materials — In the Supplementary Materials, we present the adverse events report which could not be included in the manuscript because of space limitations. The adverse events report showed the details of every adverse event reported including the description, duration, and any treatment undertaken. The relationship between the adverse events and the use of toothpaste was confirmed to be “probably irrelevant” and not correlated with toothpaste adverse reactions. [file 3764936.f1.docx]

| **Table S The list of the adverse events** | | | | | | | | | | | | | | |  |
| --- | --- | --- | --- | --- | --- | --- | --- | --- | --- | --- | --- | --- | --- | --- | --- |
| **Group** | **Number** | **Name of the adverse event** | **Description of the adverse event** | **Occurrence Time** | **End time** | **Duration** | **Severity** | **Whether belongs to serious adverse event** | **Out**  **-come** | **Measures adopted on the test toothpaste** | **Whether undertake corrective treatment** | **Whether withdraw from the trial** | **Whether caused by the test toothpaste** | **Whether belongs to the adverse reaction** | |
| B | 072 | Oral ulcer | Can’t remember the details. | 2017/10/31 | 2017/11/03 | 3 days | 1 | 0 | 1 | 1 | 0 | 0 | 5 | 0 | |
| B | 047 | Oral ulcer | 1*1mm^2^ at the buccal mucosa of the mandibular premolar area. | 2018/01/21 | 2018/01/26 | 6 days | 1 | 0 | 1 | 1 | 1 | 0 | 5 | 0 | |
| A | 060 | Cold | Nasalcongestion and runny nose. | 2017/11/22 | 2017/11/25 | 3 days | 1 | 0 | 1 | 1 | 1 | 0 | 5 | 0 | |
| B | 063 | Cold | Runny nose and sneezing. | 2017/11/20 | 2017/11/27 | 7 days | 1 | 0 | 3 | 1 | 1 | 0 | 5 | 0 | |
|  |  | Cold | Fever，sore throat and coughing. | 2018/01/22 | 2018/01/28 | 5 days | 1 | 0 | 3 | 1 | 1 | 0 | 5 | 0 | |
| B | 065 | The mandibular anterior teeth sensitive on cold and heat | The mandibular anterior teeth were sensitive when eating cold and hot food, no obvious pain, and then relieve themselves. | 2017/11/28 | 2017/12/03 | 6 days | 1 | 0 | 3 | 1 | 0 | 0 | 5 | 0 | |
| A | 070 | Pericoronitis of the wisdom tooth | Redness and swollen with inflammation around the “38” tooth. | 2017/11/30 | 2017/12/03 | 3 days | 1 | 0 | 3 | 1 | 1 | 0 | 5 | 0 | |
| A | 101 | Cold | Unspecified. | 2017/11/19 | 2017/11/26 | 7 days | 1 | 0 | 1 | 1 | 1 | 0 | 5 | 0 | |
|  |  | Sensitive teeth | Upper right posterior teeth were sensitive when brushing, no obvious pain, no medication, self-relieve. | 2017/12/08 | 2017/12/30 | 21 days | 1 | 0 | 1 | 1 | 0 | 0 | 5 | 0 | |
| B | 100 | Sensitive teeth | Sensitive to hot, cold, sweet and sour, no obvious pain, self-relieve. | 2017/11/15 | 2017/12/30 | 45 days | 1 | 0 | 1 | 1 | 0 | 0 | 5 | 0 | |
| B | 099 | Mouth and tongue mucosa numb. | It felt obvious at first, and gradually adapted. | 2017/10/24 | 2017/11/04 | 6 days | 1 | 0 | 1 | 1 | 0 | 0 | 5 | 0 | |
| A | 098 | Cold | Self-medication, not specific. | 2017/12/02 | 2017/12/03 | 1 day | 1 | 0 | 1 | 1 | 0 | 0 | 5 | 0 | |
|  |  | Sensitive teeth | Sensitive when brushing the lower left molar area; pain when touching the toothpaste and drinking cold water, (the buccal side of the “36” tooth has a wedge-shaped defect) and the pain became lighter. | 2017/12/17 | 2017/12/30 | 14 days | 1 | 0 | 4 | 1 | 0 | 0 | 5 | 0 | |
| B | 097 | Cold | Have a slight nasal congestion. | 2018/01/27 | 2018/01/28 | 1 day | 1 | 0 | 1 | 1 | 1 | 0 | 5 | 0 | |
| B | 096 | Biting uncomfortable | Felt that the biting is weak. | 2017/12/21 | 2018/01/28 | 38 days | 1 | 0 | 3 | 1 | 0 | 0 | 5 | 0 | |
| B | 90 | Lower lip ulcer | About 1*1cm^2^ in size, no medication. | 2017/12/16 | 2017/12/21 | 5 days | 1 | 0 | 1 | 1 | 0 | 0 | 5 | 0 | |
| A | 085 | Sensitive teeth | After the original desensitization toothpaste was stopped, the left upper posterior tooth were sensitive to cold for more than one month. | 2017/10/28 | 2017/12/03 | 38 days | 1 | 0 | 3 | 1 | 0 | 0 | 5 | 0 | |
|  |  | Cold | Headache uncomfortable. | 2018/01/13 | 2018/01/21 | 8 days | 1 | 0 | 3 | 1 | 1 | 0 | 5 | 0 | |
| B | 079 | Cold | Nasal congestion, fatigue and discomfort for 2 days. | 2017/11/25 | 2017/11/27 | 8 days | 1 | 0 | 1 | 1 | 1 | 0 | 5 | 0 | |
| B | 076 | Cough | Coughing, no obvious phlegm. | 2018/01/23 | 2018/01/28 | 5 days | 1 | 0 | 3 | 1 | 1 | 0 | 5 | 0 | |
| A | 118 | Gastritis | Left upper abdomen uncomfortale. | 2017/11/21 | 2017/12/03 | 12 days | 1 | 0 | 1 | 1 | 1 | 0 | 5 | 0 | |
| B | 117 | Cold | Nasal congestion, runny nose. | 2017/12/01 | 2017/12/03 | 2 days | 1 | 0 | 1 | 1 | 1 | 0 | 5 | 0 | |
| A | 114 | Whole mouth of teeth sensitive | Sensitive when eating sweet food, no special treatment. | 2017/12/01 | 2017/12/30 | 30 days | 1 | 0 | 4 | 1 | 0 | 0 | 5 | 0 | |
| B | 091 | Cold |  | 2017/12/27 | 2017/12/30 | 3 days | 1 | 0 | 1 | 1 | 1 | 0 | 5 | 0 | |
| B | 103 | Oral ulcer | The “11” tooth vestibular groove and lower lip oral ulcer are 1*1mm^2^. | 2017/12/18 | 2017/12/23 | 5days | 1 | 0 | 1 | 1 | 0 | 0 | 5 | 0 | |
| B | 029 | Oral ulcer (traumatic) | Excessive force in brushing the teeth: at the gingival mucosa at the root of mandibular central incisor, about 1*2mm in size. | 2017/12/22 | 2017/12/24 | 2 days | 1 | 0 | 1 | 1 | 0 | 0 | 5 | 0 | |
| B | 024 | Ulcer | Located at the tip of the tongue | 2017/11/15 | 2017/11/20 | 5 days | 1 | 0 | 3 | 1 | 1 | 0 | 5 | 0 | |
|  |  | Wisdom tooth pericoronitis | The periodontal of the “38” tooth is red and swollen. | 2017/11/21 | 2017/11/25 | 4 days | 1 | 0 | 3 | 1 | 1 | 0 | 5 | 0 | |
| A | 084 | Cold | Headache and runny nose. | 2017/12/02 | 2017/12/03 | 2 days | 1 | 0 | 3 | 1 | 1 | 0 | 5 | 0 | |
|  |  | Allergic dermatitis | Both hands have red spots and feel itchy. | 2017/11/29 | 2017/11/30 | 2 days | 1 | 0 | 3 | 1 | 1 | 0 | 5 | 0 | |
| B | 030 | Cold | Nasal congestion and runny nose. | 2017/11/24 | 2017/11/25 | 1 day | 1 | 0 | 4 | 1 | 0 | 0 | 5 | 0 | |
| B | 036 | Diarrhea and low fever | Diarrhea after eating beef, causing low fever. | 2018/01/08 | 2018/01/10 | 2 days | 1 | 0 | 1 | 1 | 1 | 0 | 5 | 0 | |
| A | 032 | Cold | Self-reported headache, runny nose for two or three days, not specific. | 2017/11/20 | 2017/11/25 | 5 days | 1 | 0 | 4 | 1 | 1 | 0 | 5 | 0 | |
| B | 009 | Cold | Coughing, runny nose. | 2017/11/20 | 2017/11/28 | 8 days | 1 | 0 | 3 | 1 | 0 | 0 | 5 | 0 | |
| B | 004 | Feeling numbness of the oral mucosa | At the inner mucosa of the lower lip, lasted for a few minutes after gargling. | 2017/10/21 | 2017/11/25 | 35 days | 1 | 0 | 3 | 1 | 0 | 0 | 5 | 0 | |
| B | 008 | Ulcer | There is a soybean-sized ulcer in the vestibular groove of the “46” tooth. | 2017/11/16 | 2017/11/25 | 9 days | 1 | 0 | 3 | 1 | 0 | 0 | 5 | 0 | |
| A | 023 | Cold | Coughing, runny nose. | 2017/12/24 | 2017/12/24 | 7 hours | 1 | 0 | 4 | 1 | 0 | 0 | 5 | 0 | |
| A | 045 | Herpes | There are two spots of herpes on the lower lip. | 2017/11/21 | 2017/12/25 | 4 days | 1 | 0 | 3 | 1 | 0 | 0 | 5 | 0 | |
|  |  | Cold | Coughing, headache. | 2018/01/05 | 2018/01/12 | 7 days | 1 | 0 | 3 | 1 | 1 | 0 | 5 | 0 | |
| A | 049 | Cold | Sore throat, stuffy nose. | 2017/11/18 | 2017/11/09 | 1 day | 1 | 0 | 3 | 1 | 0 | 0 | 5 | 0 | |
| A | 050 | Cold | Coughing. | 2017/12/10 | 2017/12/17 | 7 days | 1 | 0 | 3 | 1 | 0 | 0 | 5 | 0 | |
| A | 053 | Cold | Headache. | 2017/12/16 | 2017/12/23 | 7 days | 1 | 0 | 3 | 1 | 0 | 0 | 5 | 0 | |
| A | 061 | Cold | Coughing, sore throat. | 2017/11/20 | 2017/11/24 | 4 days | 1 | 0 | 1 | 1 | 0 | 0 | 5 | 0 | |
|  |  | Oral ulcer | Located in the left cheek around the “26” tooth), about 2*2mm^2^ in size, self-healing. | 2017/12/24 | 2017/12/27 | 3 days | 1 | 0 | 3 | 1 | 0 | 0 | 5 | 0 | |
| B | 062 | Cold | Dizziness, headache and runny nose. | 2017/11/15 | 2017/11/22 | 7 days | 1 | 0 | 1 | 1 | 0 | 0 | 5 | 0 | |
|  |  | Recurrent aphthous ulcer | Ulcer for 1 week, a long-legged ulcer on the left cheek (7 mm); a round ulcer (3 mm) next to the left uvula. | 2017/12/21 | 2017/12/28 | 7 days | 1 | 0 | 3 | 1 | 1 | 0 | 5 | 0 | |
| A | 067 | Bronchitis | Catch a cold, coughing. | 2017/11/11 | 2017/11/21 | 10 days | 1 | 0 | 1 | 1 | 1 | 0 | 5 | 0 | |
| B | 069 | Cold | Headache, stuffy nose. | 2018/01/20 | 2018/01/23 | 3 days | 1 | 0 | 3 | 1 | 1 | 0 | 5 | 0 | |
| B | 071 | Cold | Sneezing and coughing. | 2017/11/10 | 2017/11/13 | 3 days | 1 | 0 | 1 | 1 | 0 | 0 | 5 | 0 | |
| A | 073 | Cold | Low fever, headache and coughing. | 2018/01/05 | 2018/01/12 | 7 days | 1 | 0 | 3 | 1 | 1 | 0 | 5 | 0 | |
| B | 102 | Cold | Nasal congestion, runny nose and headache. | 2018/01/27 | 2018/01/28 | 2 days | 1 | 0 | 3 | 1 | 1 | 0 | 5 | 0 | |
| Notes: (1) Severity: 1: light; 2: medium; 3: heavy. (2) Whether belongs to serious adverse event: 1: yes; 0: no. (3) Outcomes: 1: disappeared; 2: sequelae; 3: relieved; 4: sustained; 5: died. (4) Measures adopted on the test toothpaste: 1: continue to use; 2: reduce the dose; 3: discontinue use; 4: completely deactivated. (5) Whether undertake corrective treatment: 1: Yes; 0: No. (6) Whether withdraw from the test: 1: Yes; 0: No. (7) Whether caused by the test toothpaste: 1: affirmative; 2: most likely; 3: possible; 4: suspicious; 5: impossible. Affirmative, very likely, possible, and suspicious 4 are considered adverse reactions of toothpaste. (8) Whether belongs to an adverse reaction of toothpaste: 1: yes; 0: no. | | | | | | | | | | | | | | |  |
